# The impact of single nucleotide polymorphism selection on prediction of genomewide breeding values

**DOI:** 10.1186/1753-6561-3-s1-s13

**Published:** 2009-02-23

**Authors:** Kacper Żukowski, Tomasz Suchocki, Anna Gontarek, Joanna Szyda

**Affiliations:** 1 Institute of Animal Genetics, Wrocław University of Life and Environmental Sciences, Wrocław, Poland; 2 Institute of Natural Sciences, Wrocław University of Life and Environmental Sciences, Wrocław, Poland

## Abstract

The study focuses on the impact of different sets of single nucleotide polymorphisms (SNPs) selected from the available data set on prediction of genomewide breeding values (GBVs) of animals. Correlations between breeding values estimated as additive polygenic effects (EBVs) and GBVs as well as correlations between true breeding values (TBVs) and GBVs are used as major criteria for the comparison of different SNP selection schemes and GBV estimation models.

The analysed data is the simulated data set from the XII QTL Workshop. In the analysis five different SNP data sets are considered. For prediction of EBVs a standard mixed animal model is applied, whereas GBVs are defined as the sum of additive effects of SNPs estimated for the different SNP data sets using model 1 with fixed SNPs effects, model 2 with fixed SNPs effects and a random additive polygenic effect, model 3 with a random effects of uncorrelated SNP genotypes.

The additive polygenic and residual variance components estimated by the EBV model amount to 1.36 and 3.12, respectively. Differences between models are expressed by comparing the ranking of individuals based on EBV and on GBV and by correlations. Among 100 individuals with the highest EBVs, depending on a model and a data set, there are only between 11 and 37 individuals with the highest GBVs. The highest correlation between GBV and EBV amounts to 0.787 and is observed for model 3 with 3,328 SNPs selected based on their minor allele frequency, the lowest correlation of 0.519 is attributed to model 2 with 300 SNPs. Correlations between GBV estimates obtained from different models with the same number of SNPs range between 0.916 and 0. 998, whereas correlations between different SNP data sets using the same model fall under 0.850.

These results indicate that successful application of high throughoutput SNP genotyping technologies for prediction of breeding values is a very promising approach, but before the method can be routinely applied further methodological improvements regarding model construction and SNP selection are required.

## Background

The idea behind using high throughoutput single nucleotide polymorphism (SNP) microarray technology in cattle breeding industry is based on the assumption that the additive genetic merit of animals (mainly bulls) can be accurately predicted based on their genotypes at many SNPs. This study focuses on the impact of different sets of SNPs selected from the available data set of 6,000 SNPs on prediction of GBVs of animals. Correlations between breeding values estimated as additive polygenic effects (EBVs) using a standard mixed animal model, and GBVs are used as a major criterion for the comparison of different SNP selection schemes and different GBV estimation models.

## Methods

The analysed data is the simulated data set from the XII QTL Workshop, consisting of 5,865 individuals from seven generations, divided into (i) a group of 4,665 animals from generations 1–4 for which both phenotypes and genotypes are available, (ii) a group of 1,200 animals from generations 5–7 for which only genotypes are available. Phenotypes represent a quantitative trait, while genotypes represent 6,000 SNP markers evenly distributed every 0.1 cM over six chromosomes. In our analysis five different SNP data sets are considered. They comprise:

- a set of all available 6,000 SNPs (SNP6000),

- a set of 3,328 SNPs selected based on their estimated minor allele frequency (MAF) using the condition: MAF ≥ 0.3 (SNP3328),

- a set of 1,200 SNPs selected as every 5^th ^SNP out of the available set (SNP1200),

- a set of 600 SNPs selected as every 10^th ^SNP out of the available set (SNP600),

- a set of 300 SNPs selected as every 20^th ^SNP out of the available set (SNP300).

For prediction of EBVs a standard mixed animal model is applied: **y **= *μ *+ **Zα **+ **e**, where **y **is a vector of phenotypic values, μ is the overall mean, $\alpha \sim N\left(0,A\sigma_{\alpha }^{2}\right)$ is a vector of random additive polygenic effects of animals with a covariance matrix given by the numerator relationship matrix (**A**) and the component of the additive polygenic variance $\sigma_{\alpha }^{2}$, and $e\sim N\left(0,I\sigma_{e}^{2}\right)$ is a vector of residuals. GBVs are defined as the sum of additive effects of SNPs, estimated from different SNP data sets defined above using the following models:

- (1) **y **= *μ *+ **Xq **+ **e**, where **q **(N_SNP _× 1) is a vector of fixed additive SNP effects with the corresponding design matrix **X **with score 0, 1, or 2 for an SNP genotype 11, 12, or 22 respectively, N_SNP _is the number of SNPs considered and other model parameters are defined as above.

- (2) **y **= *μ *+ **Xq **+ **Zα **+ **e**, with all the parameters defined as above.

- (3) **y **= *μ *+ **Zq **+ **e**, where $q\sim N\left(0,I\sigma_{\alpha }^{2}\right)$ is a vector of random SNP effects with the corresponding design matrix **Z **with score 0, 1, or 2 for an SNP genotype 11, 12, or 22 respectively.

Note that EBVs and GBVs are estimated for the 4,665 animals from the first four generations. The estimation of parameters of all the mixed models was based on solving the mixed model equations (MME, [1]) while effects in model 1 were estimated using the least squares approach. The DFREML package [2] was used for the estimation of parameters and variance components of the EBV model, whereas the parameters of GBV models (model 1–3) were estimated using R programmes. For models 1–3 residual and additive polygenic variance components were assumed as known and were set with the estimates obtained from the EBV model. Due to too high memory requirements for building an inverse of the coefficient matrix of MME, we were unable to estimate parameters of models 2 and 3 for the data set with all SNPs.

## Results and discussion

### Variance components

The additive polygenic and residual variance components estimated by the EBV model amount to 1.36 and 3.12, respectively, which results in a heritability of 0.30.

### Ranking of individuals based on EBVs and on GBVs

**Figure 1:**
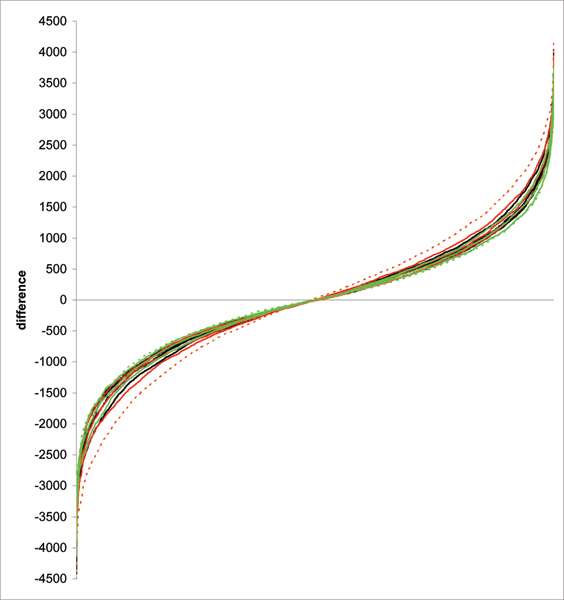
**Differences in ranking of individuals based on EBV and on GBVs**. Individual differences in ranks based on EBV and different GBV models and for different SNP data sets, calculated for animals from the first four generations and sorted in ascending order. Model 1 is represented by black curves, model 2 – by red curves, and model 3 – by green curves. The best (lowest differences) and the worst (highest differences) models are represented by dashed curves.

Differences between the models expressed in the similarity in ranking of 100 individuals with the highest GBV are summarised in Table 1. When the ranking based on EBV is treated as a basis, the highest ranking similarity is observed for GBV_SNP6000 _of model 1 which has 41% correspondence with the 100 individuals with the highest rank based on EBV. The lowest similarity of 11% is observed for GBV_SNP300 _of model 2. In general, for a given number of SNPs model 2 has mostly the lowest number of individuals in the top 100 ranking based on EBV, while model 3 – mostly the highest. Consequently, when differences in ranking are compared on an individual level, the smallest differences are observed for model 3 with 3328 SNPs and highest differences – for model 2 and 300 SNPs (Figure 1). However in general, individual differences in ranks are similar across models and SNP data sets.

**Table 1:** Differences in top 100 ranking of individuals.

	**Model 1**	**Model 2**	**Model 3**
GBV_SNP6000_	41	NA	NA
GBV_SNP3328_	36	35	35
GBV_SNP1200_	30	32	37
GBV_SNP600_	26	18	31
GBV_SNP300_	23	11	25

The number of 100 best individuals as ranked by GBV models contained within the set of 100 best individuals as ranked by the EBV model calculated for individuals from the first four generations. GBV are calculated for different SNP data sets, as indicated in subscripts. NA, not available.

### Correlations between EBV and GBV

Correlations between EBVs and GBVs calculated from the three models and different SNP data sets for individuals from the first four generations are presented in Table 2. Generally, correlations between GBV and EBV are far from one and they decrease with the decreasing number of SNPs considered in a model. The highest correlation is estimated for model 3 and SNP3328 amounting to 0.787, the lowest correlation of 0.519 is attributed to model 2 and SNP_300_. Note, that in model 3 a relatively large variance parameter of $\sigma_{\alpha }^{2}$ was assumed for the SNP effect, while a common approach to modelling random SNP effects is to apply the variance estimator of $\frac{\sigma_{\alpha }^{2}}{N_{SNP}}$. However, in terms of correlations between GBV and EBV, there was practically no difference between the models assuming the two different variance estimators (results not presented).

**Table 2:** Correlation between EBV and GBV.

	**Model 1**	**Model 2**	**Model 3**
GBV_SNP6000_	0.761	NA	NA
GBV_SNP3328_	0.750	0.758	0.787
GBV_SNP1200_	0.742	0.714	0.777
GBV_SNP600_	0.720	0.643	0.745
GBV_SNP300_	0.665	0.519	0.694

Correlations between EBV and GBV calculated for individuals from the first four generations and for different SNP data sets, as indicated in subscripts. NA, not available.

In the paper of Meuwissen *et al*. [3], which was a pioneering in the filed of using multiple SNPs for the prediction of GBV, a similar correlation of 0.73 between true and predicted GBV, based on a random SNP haplotype effects, was reported. However, using fixed SNP effects resulted in a correlation as low as 0.32 – lower than the correlation in our study if at least 1200 SNPs are considered. Much higher correlations of 0.95 between true additive genetic values and GBVs estimated by a model with random SNP genotype effects and a model with a random additive polygenic effect with SNP effect modelled by a kernel function, were observed by Gianola *et al. *(2006) [4], but for the favourable conditions of unrelated individuals, no correlations between SNPs, and all 100 loci determining a trait fitted into the model. Similar correlations were also reported by Habier *et al*. [5].

### Correlations between GBVs

**Figure 2:**
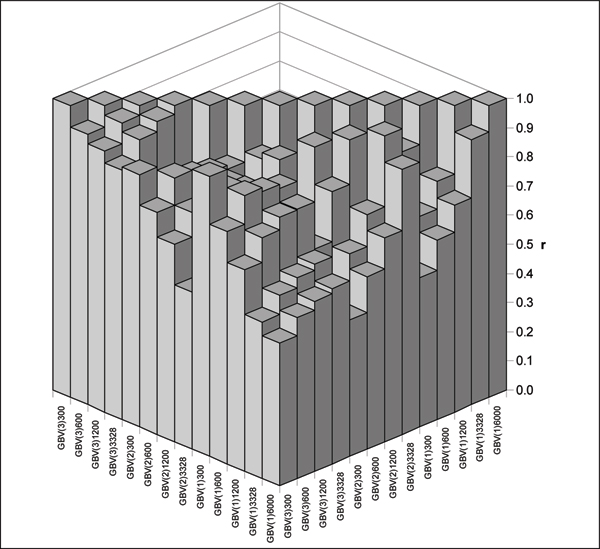
**Correlations between GBVs**. Correlations (r) between GBVs estimated by different models and for different SNP data sets. Models are indicated in parentheses, followed by the number of SNPs used.

A general overview of correlations between different GBVs is given in Figure 2. Correlations vary considerable from 0.99 between GBV_SNP3328 _for model 1 and model 2, as well as between GBV_SNP1200 _also for models 1 and 2 to as low as 0.47 between GBV_SNP6000 _for model 1 and GBV_SNP300 _for model 2. In general correlations between predicted GBVs resulting from models using the same number of SNPs are relatively high exceeding 0.80 (except two correlations involving GBV_SNP3328 _for model 3). Correlations between GBV estimates obtained from the same model, but using different N_SNP _are lower, generally falling under 0.70 for models 1 and 2 and somewhat higher – from 0.97 to 0.85 for model 3.

### Residual variances

Table 3 summarises residual variances resulting from each of the GBV estimation models applied. The lowest value of 0.20 obtained for model 1 and 6000 SNPs indicates overfitting of the model. For data sets with the lower number of SNPs considered there are only minor differences in residuals variance, however model 3 always results in the highest values.

**Table 3:** Residual variances.

	**Model 1**	**Model 2**	**Model 3**
GBV_SNP6000_	0.20	NA	NA
GBV_SNP3328_	0.91	0.64	3.00
GBV_SNP1200_	2.29	2.29	3.11
GBV_SNP600_	2.82	2.82	3.23
GBV_SNP300_	3.31	3.31	3.45

Residual variances calculated for the GBV estimation models and for different SNP data sets, as indicated in subscripts. NA, not available.

Summarising, each of methods applied in the present study has its drawbacks: the mixed animal model unifies the additive genetic background and does not properly account for the existence of QTL along the genome, model 1, with the increasing number of SNPs included, suffers problems related to over fitting, models 1 and 3 do not use information on relationship among individuals, in model 2 the additive polygenic relationships are given too much emphasis since the corresponding variance component was not estimated for this model, but simply assumed as known and equal to the variance component of a pure polygenic model without SNPs. The number of fitted SNPs not only influences on the estimates of GBV, but also the feasibility of computations – that is why it should be treated with caution. Although the highest EBV-GBV correlations are obtained for data a set with all 6000 SNPs, similar values are observed using a bit more than a half of SNPs selected based on MAF.

## Conclusion

The most important result of this study, also reported by other authors [3,5], are overall low correlations between EBVs and GBVs which indicate that both quantities cannot be regarded as describing the same genetic background. The correlations between TBVs and GBVs are even lower [6].

Summarising, relatively simple models applied in this study are not stable enough (e.g. robust towards the number of fitted SNPs, poorly correlated with EBV) to be used for routine national genetic evaluation of dairy cattle, especially if the EBVs estimated using a classical method are to be regarded as the desired selection criterion. On the other hand practical application of more sophisticated methods is hampered by computational problems. Although successful using of high throughoutput SNP genotyping technologies for prediction of breeding values is a very promising approach, before the method can be routinely applied, further methodological improvements regarding model construction and SNP selection procedures are needed.

## Competing interests

The authors declare that they have no competing interests.

## Authors' contributions

KŻ and TS wrote computer programs and performed statistical analysis of data, AG edited raw data and selected SNPs, JS is responsible for the scientific concept of the analysis, preparation of the manuscript and for coordination of the project.
